# Photocatalytic dye degradation and antimicrobial activities of Pure and Ag-doped ZnO using Cannabis sativa leaf extract

**DOI:** 10.1038/s41598-020-64419-0

**Published:** 2020-05-12

**Authors:** Ankush Chauhan, Ritesh Verma, Swati Kumari, Anand Sharma, Pooja Shandilya, Xiangkai Li, Khalid Mujasam Batoo, Ahamad Imran, Saurabh Kulshrestha, Rajesh Kumar

**Affiliations:** 1 School of Physics and Materials Science, Shoolini University of Biotechnology & Management Sciences, Bajhol-Solan, HP 173212 India; 2 School of Applied Science and Biotechnology, Shoolini University of Biotechnology & Management Sciences, Bajhol-Solan, HP 173212 India; 3 School of Chemistry, Shoolini University of Biotechnology & Management Sciences, Bajhol-Solan, HP 173212 India; 4 Ministry of Education Key Laboratory of Cell Activities and Stress Adaptations, School of Life Science, Lanzhou University, Tianshuinanlu #222, Lanzhou, 730000 Gansu Province P.R. China; 5 King Abdullah Institute for Nanotechnology, King Saud University, P.O. Box-2455, Riyadh, 11451 Saudi Arabia

**Keywords:** Materials science, Nanoscale materials

## Abstract

A facile green route has been employed for the synthesis of ZnO and Ag-doped ZnO using Cannabis sativa as a reducing and stabilizing agent. The as-synthesized nanoparticles were characterized and tested for photocatalytic dye degradation and antimicrobial activity. The results suggested that nanoparticles have shown antimicrobial activity against different human pathogenic bacteria (*Escherichia coli, Klebsiella pneumonia*, MRSA, *Pseudomonas aeruginosa, Salmonella typhi*, *Staphylococcus aureus*) and fungal strains (*Fusarium* spp. and *Rosellinia necatrix)*. Ag-doped nanoparticles comparatively have shown better removal Congo red and methyl orange under visible light. Therefore, green synthesized nanoparticles could have beneficial applications in environmental science and biological field.

## Introduction

Nanotechnology plays an essential role in modern material science, capable of diverse novel applications in biomedical sciences, pharmaceutical sciences, medicine, nutrition, energy etc^1^. Some pertinent materials exhibit new and enhanced physiochemical and biological properties with distinct functionalities due to their nano level size (1–100 nm)^2^. The inorganic nanoparticles such as Ag, Au, CuO, TiO_2_ and ZnO have profound applications in almost every field because they are easy to prepare, inexpensive and safe for the human being. However, ZnO nanoparticles are in the scientific spotlight because of their unique properties such as semiconducting property^3^, piezoelectric property, optical property, antibacterial property^4^, anti-fungal and wound healing property and UV filtering property, high catalytic and photochemical activity^5,6^. It is significant material in the ceramic industry because of its hardness and rigidity, while, its low toxicity, biocompatibility and biodegradability make it an element of interest for biomedicine and pro-ecological systems such as cosmetics^7,8^. These nanoparticles interact with cell biomolecules in a unique manner and facilitate the physical transfer of nanoparticles into inner cellular structures. Nanomaterials have high surface reactivity due to their large surface area^9^. ZnO possesses high photocatalytic efficiency among all inorganic photocatalytic materials and has a high response to UV light which significantly activates the interaction of ZnO with bacteria. ZnO nanoparticles show the phototoxic effect which produces reactive oxygen species, essential for biological applications^10–13^. The introduction of selective dopant on ZnO nanoparticles significantly shown enhances the photocatalytic activity with improved Z-scheme^14,15^ mechanism which enhances visible light photocatalytic activity^16–19^ and increases the antimicrobial activity^20^. It has been shown that there are many physical, chemical and biological routes to synthesise nanoparticles. However, recently increasing awareness towards green chemistry and other natural processes have led to the development of an eco-friendly approach for the synthesis of nanoparticles^21^. Green synthesis of nanoparticles is an approach that effectively connects nanotechnology to biotechnology. In green synthesis route, nanoparticles are obtained with minimum defects and with homogeneous chemical composition. It has been known that plants have the property to reduce metal ions so the extract of plants, microorganisms, viruses or their by-products (proteins and lipids) are widely used in greener route of synthesis^13,22,23^. These natural strains and plant extracts secrete some phytochemicals that act as both reducing and capping or stabilization agent. Now, the question arises that why this greener route of synthesis is gaining so much importance nowadays? The answer may include the following reasons: (a) unique properties of nanoparticles obtained from green synthesis over physic-chemical methods; (b) nanoparticles synthesised by physic-chemical routes have some problems with it like toxicity, hazardous by-product generation and the imperfection of surface structure etc.; (c) nanoparticles synthesised from biological activity have diverse nature, excellent stability and appropriate dimensions as they are synthesised using one-step procedure; (d) green synthesis provides natural reducing, capping and stabilizing agents over the expensive chemicals; and (e) physic-chemically synthesised nanoparticles may be dangerous to human health or the health of our environment^24–27^. The biological approach to synthesise nanoparticles appears to be cost-effective over to the conventional physical and chemical routes.

ZnO nanoparticles have been reposted in different morphologies such as nanoflower, a nanobelt, nanorods^28^, nanowires^29^, microspheres^30,31^, nanoflake etc. and depend upon the nature of synthesis^28,31,32^. However, green synthesis of ZnO nanoparticles allows large-scale production with less additional impurities. ZnO nanoparticles have been reported to be synthesised from many plant extracts such as seaweeds of Gulf of Mannar^33^, *Aeromonas hydrophila*^34^, Trifoliate orange^6^, *Parthenium hysterophorus*^35^, *Calatropis gigantea*^36^, *Citrus aurantifolia*^37^, *Ocimum sanctum*^38^, Maple leaf^39^, *Tamarindus indica* (L.)^40^, *Solanum nigrum*^41^ etc.

*Cannabis* is a species of the *Cannabinaceae* family of plants and also known as weed hemp, Ganja, Hashish, reefer, marijuana, grass. Originating from Central Asia, cannabis is one of the oldest psychoactive plant known to man, but it has also been used all over the world either as a medicinal plant or as a source of fibres and food^42^. In India, marijuana was being used for medicinal purposes since 1500 B.C., in Greece since 70 A.D., and by the early 1500 s in Europe. Over centuries, it continued to grow around the world as people used it for different reasons. Cannabis may increase the effects of other drugs that cause drowsiness, including antidepressants, alcohol, antihistamines, sedatives (used to treat insomnia), pain relievers, anxiety medicines, seizure medicines, and muscle relaxants. The Cannabis plant and its products consist of an enormous variety of chemicals. There are over 60 chemicals in cannabis, which may have medical uses. Since a couple of years, there is an increasing number of scientific shreds of evidence showing the efficiency of cannabinoids in the treatment of Parkinson disease, Tourette’s syndrome, antiemetic effect, analgesia, epilepsy, neurological conditions, multiple sclerosis and appetite disorders^43^. The physiology of *C. sativa* is already well understood. It is known that the highest concentration of active compounds is concentrated in inflorescent; therefore, it is surprising that in most studies assessing antimicrobial properties of cannabis, leaves and seeds are usually used to prepare extracts. The leaves were once used in bandages and a relaxing non-psychoactive herbal tea can be made from small cannabis stems. *Cannabis sativa* is a source of various biologically active compounds. The main compounds that are found in this plant are cannabinoids, flavones, and terpenes. In 2016, Khan *et al*., revealed that secondary metabolites i.e., terpenoid plays a significant role as a reducing agent in NPs synthesis^44^.

The present work reports the synthesis of pure ZnO and Ag-doped ZnO using *Cannabis sativa* leaf extract as a reducing agent. The nanoparticles were studied for photocatalytic dye degradation and antimicrobial activities.

## Experimental Details

### Materials

The chemicals, zinc acetate, sodium hydroxide and silver nitrate were purchased from sigma company, while the *Cannabis sativa* leaves were collected from the Himalayan region.

### Preparation of Cannabis leaf extract

The aqueous extract of *Cannabis sativa* was prepared using a water bath. 10 g of shade-dried *C. sativa* leaves were crushed and added to 100 ml of deionized water in 250 ml conical flask. The hot water bath was set at 60 °C for 12 hrs. The solution was allowed to cool down and filtered using filter paper. The prepared cannabis leaf extract was further used for the synthesis of nanoparticles.

### Preparation of nanoparticles

For the preparation of Zinc Oxide nanoparticles, 0.5 molar solution of zinc acetate was prepared in 100 ml deionized water. The solution was magnetically stirred and maintained at 40°C temperature. 5 ml solution of 0.2 molar sodium hydroxide was added to the previous solution and stirred for 30 minutes. 10 ml of *Cannabis sativa* leaf extract was added to the solution and stirred for around 2 hrs and white coloured precipitates were observed in the solution. Similarly, for the preparation of silver-doped zinc oxide nanoparticles, 0.05 molar solution of silver nitrate in 50 ml was added to the 100 ml solution of zinc acetate and stirred for 10 minutes at a constant temperature of 40 °C.10 ml solution of 0.2 molar sodium hydroxide was added and allowed to stir for 50 minutes. 12 ml of *Cannabis sativa* leaf extract was added to the previous solution and stirred for two hours. Black coloured precipitates were observed when kept still for a few hours. Precipitates were then collected using a centrifuge and washed 5 times with deionized water for the removal of impurities. The samples were then dried at 80°C for 12 h. Method opted for the synthesis was from previous reported work^45^. The obtained nano-powders were then characterized for various structural, morphological, optical and antimicrobial properties.

## Results an Discussions

### Structural and morphological studies

The phase formation and purity of compounds were verified with X-ray diffraction (XRD) technique. X-ray powder diffractometer (Rigaku Minifiex 600, Japan) with CuKα radiation (λ = 1.5405 Å) in an extensive range of Braggangles 2θ (20°≤2θ ≤ 60°) at a scanning rate of 2° min^−1^ at room temperature. Surface morphology of the samples was studied using FESEM from Hitachi SU 8010 using an operating voltage of 5 kV. XPS study was carried using Nexsa base model (make: Thermo fisher scientific). TEM study along with EDS and elemental mapping was employed using FP 5022/22-Tecnai G2 20 S-TWIN (make: FEI company of USA).

### Antibacterial studies

For antibacterial studies, the microbial strain *Escherichia coli* (MTCC 82), *Klebsiella pneumonia* (MTCC-39), MRSA (Methicillin-resistant Staphylococcus aureus Standard strain-CA 05 SCCmec Type IV), *Pseudomonas aeruginosa* (MTCC 2453), *Salmonella typhi* (MTCC 734), *Staphylococcus aureus* (MTCC 96), were obtained from parasitology laboratory, and for antifungal studies, the strains *Fusarium* spp., *Sclerotinia sclerotiorum* and *Rosellinia necatrix* were obtained from Molecular plant-microbe interaction laboratory of Shoolini University of Biotechnology and Management Sciences, Solan, India.

### Antibacterial Assay

Antimicrobial activity was done with the help of agar well diffusion method^46^. 100 µl of the bacterial inoculums was spread over plates containing nutrient agar and then 6 mm wells were created with the help of puncture on the plates and check the antimicrobial activity by taking 100 $\mu {\rm{l}}$ of nanoparticle extracts against all pathogenic bacteria. Two controls were included in the test i.e., the antibiotic ampicillin, which was considered as positive control and 10% DMSO which was taken as a negative control. The plates were kept in the incubator for 18–24 hrs at 37 °C. All the tests were performed in triplicate. The inhibition zones obtained around the wells were measured. The inhibition zones were measured by taking the amount of 100 µl of nanoparticle extract in a different well. The antibiotic ampicillin (+ve control) showed the inhibition zone by taking the amount of 10 µl at the concentration of 100 mg/ml. 10% DMSO (−ve control) showed no zone against all bacteria.

### Antifungal Assay

Nanoparticles extract were prepared at concentrations 1%, were added in 25 ml of sterilized potato dextrose agar in petri plates. A 6 mm diameter of the actively growing mycelium disc of the pathogen of 6–7 day old culture was placed in the center of the petri dish. Plates without extract served as the negative control. Plates were incubated at 25 °C. Radial growth of mycelium was measured after seven days of incubation. The growth results were compared with the negative control. The experiment was repeated three times, and the mean of the readings was taken for further calculations. The percent inhibition of the fungus in the experiment was calculated using the following formula;

1 $$L=\left[\frac{(C-T)}{C}\right]\times 100$$

Where L is the percent inhibition; C is the colony radius in the control plate, and T is the radial growth of the pathogen in the presence of nanoparticles extracts^47^.

### Photodegradation analysis

The photocatalytic activity of ZnO and Ag doped ZnO was evaluated for the photodecomposition of congo red and methyl orange listed in Table 1 under solar light irradiation. To confirm degradation process, small volume of aliquot was taken and centrifuged for 5 min to completely remove nanoparticles. The concentration of azo dyes was detected using UV-Vis spectrophotometer. The self-degradation of congo red and methyl orange under solar irradiation is not significant.

**Table 1:** Presents Chemical properties of dyes.

Sr. No.	Common Name/Molecular formula	Chemical Structure	Molecular weight	Type
1.	Congo red (C_32_H_22_N_6_Na_2_O_6_S_2_)		696.7 g/mol	Anionic
2.	Methyl Orange (C_14_H_14_N_3_NaO_3_S)		327.3 g/mol	Anionic

## Results and Discussion

### XRD Analysis

**Figure 1:**
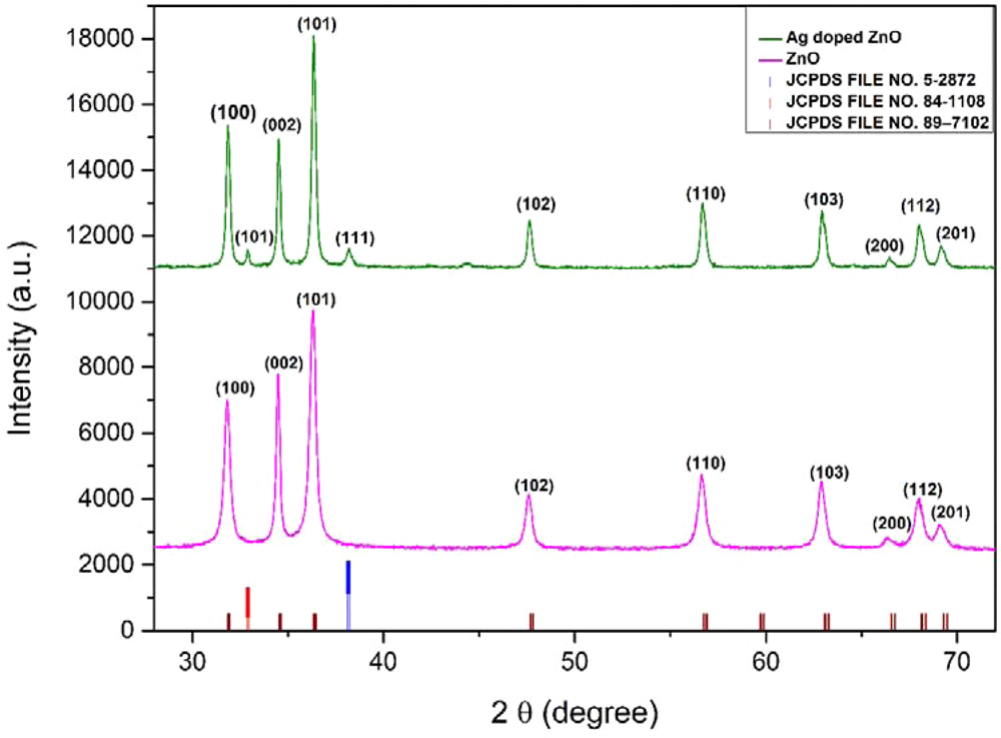
Presents XRD pattern of ZnO and Ag-ZnO nanoparticles.

The Phase identification and purity of the samples were confirmed by X-ray diffraction measurement. The XRD patterns of the pure and Ag-doped ZnO matched with phases of Zinc Oxide and AgO as a secondary phase in good agreement with COD ID: 2300112 (ZnO) and COD ID: 4318188 (AgO) using MATCH software. Pure and silver doped Zinc oxide nanoparticles exhibited the characteristic peaks of hexagonal wurtzite structure revealing ZnO dominating structure in Ag-doped ZnO corresponding to reflection planes (100), (002), (101), (102), (110), (103), (200), (112) and (201)in good agreement with JCPDS FILE NO. 89–7102, as shown in Fig. 1. Two additional peaks with low intensities were observed in XRD pattern of Ag-doped ZnO at 33.01° and 38.20° corresponding to the AgO(JCPDS FILE NO. 84–1108) and elemental Ag(JCPDS FILE NO. 5–2872)^48^.

The particle size listed in Table 2 was calculated using the Scherrer formula^49^,

2 $$D=\frac{0.9\lambda }{\beta Cos\theta }$$

Where D is the average crystallite size, λ is the X-ray wavelength, θ is the Bragg angle, and β is the FWHM of the experimental data. Dislocation density S was calculated using crystallite size (D) which represents the amount and defects present in the crystal system, given by the formula^50^ and listed in Table 1:-

3 $$S=\frac{1}{{D}^{2}}$$

**Table 2:** Presents Parameters analyzed by Scherrer method, William-Hall method and Rietveld analysis.

Compound	ZnO	Ag-ZnO
Structure	Hexagonal	Hexagonal
Space group	P63mc	P63mc
**Lattice parameters**		
a = b (Å)	3.2523	3.2507
c (Å)	5.2096	5.2081
Volume (Å)^3^	47.723	47.664
**Atomic coordinates**		
**Zn**		
$x$	0.3333	0.3333
$y$	0.6667	0.6667
$z$	0.0000	0.0000
**O**		
$x$	0.3333	0.3333
$y$	0.6667	0.6667
$z$	0.3819	0.3819
**Refined parameters**		
${\chi }^{2}$	2.578	2.635
${R}_{p}$	11.7	12.6
${R}_{wp}$	11.6	12.6
${R}_{e}$	7.01	7.75
Particle size (Scherrer method) (nm)	21.05	30.13
Particle size (W-H method) (nm)	23.32	35.15
Strain(**ε**) and Dislocation density	0.00229, 0.0022	0.00677, 0.0011

The William Hall method was employed for determining the crystallite size and lattice strain for both ZnO and Ag-doped ZnO using the William Hall equation given below^51^:-

4 $$\beta Cos\theta =\frac{k\lambda }{D}+4\varepsilon Sin\theta $$

Where β is full width at half maximum (FWHM), D is the crystallite size, and ε is the strain

Plotting βcosθ against 4εsinθ from the linear fit of the data, we determined the lattice strain from the slope and crystallite size using the intercept as listed in Table 2.

The crystallite sizes obtained from Sherrer method are smaller in comparison to William-hall method because Sherrer method measures the cohesion length of the X-rays any defects and vacancies will cause the measured size to be lower than the actual size whereas William-hall method takes micro-strain into consideration.

**Figure 2:**
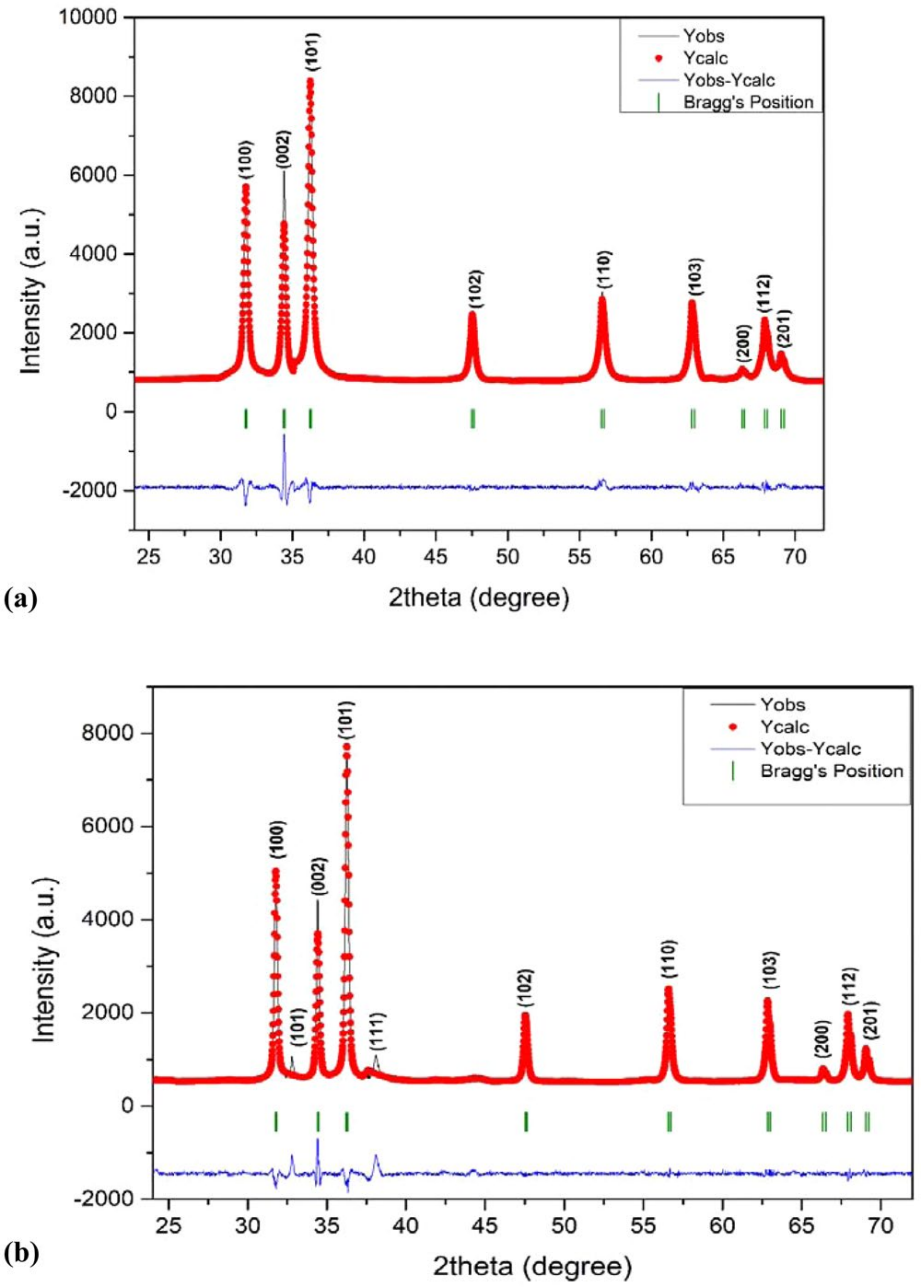
(**a,b**) Presents Rietveld analysis of (**a**) ZnO and (**b**) Ag-ZnO nanoparticles.

Rietveld refinement of both the XRD patterns was done using Full prof program with space group P63mc.The background was fitted with linear interpolation, and peak patterns were described by pseudo-Voigt profiles. First, global parameters such as background, instrumental and scale factors were refined and further cell parameters, FWHM parameters, shape parameters, preferred orientation and atomic positions were refined in sequence. There is a slight decrease in the lattice parameters of Ag-doped ZnO but lack of peak shift in the experimental data suggests that there may be the segregation of AgO and Ag species on the grain boundaries of ZnO crystal or an inadequate amount of silver atoms would have incorporated in ZnO crystal^52^. Figure 2(a,b) shows the Rietveld analysis of ZnO and Ag-ZnO nanoparticles, respectively.

It can be seen in Fig. 2 that the observed and calculated profiles are in good resemblance to each other and all calculated parameters along with reliability factors R_factors_ (R_p_, R_wp_, R_exp_, R_b_) as listed in Table 2.

### XPS Analysis

**Figure 3:**
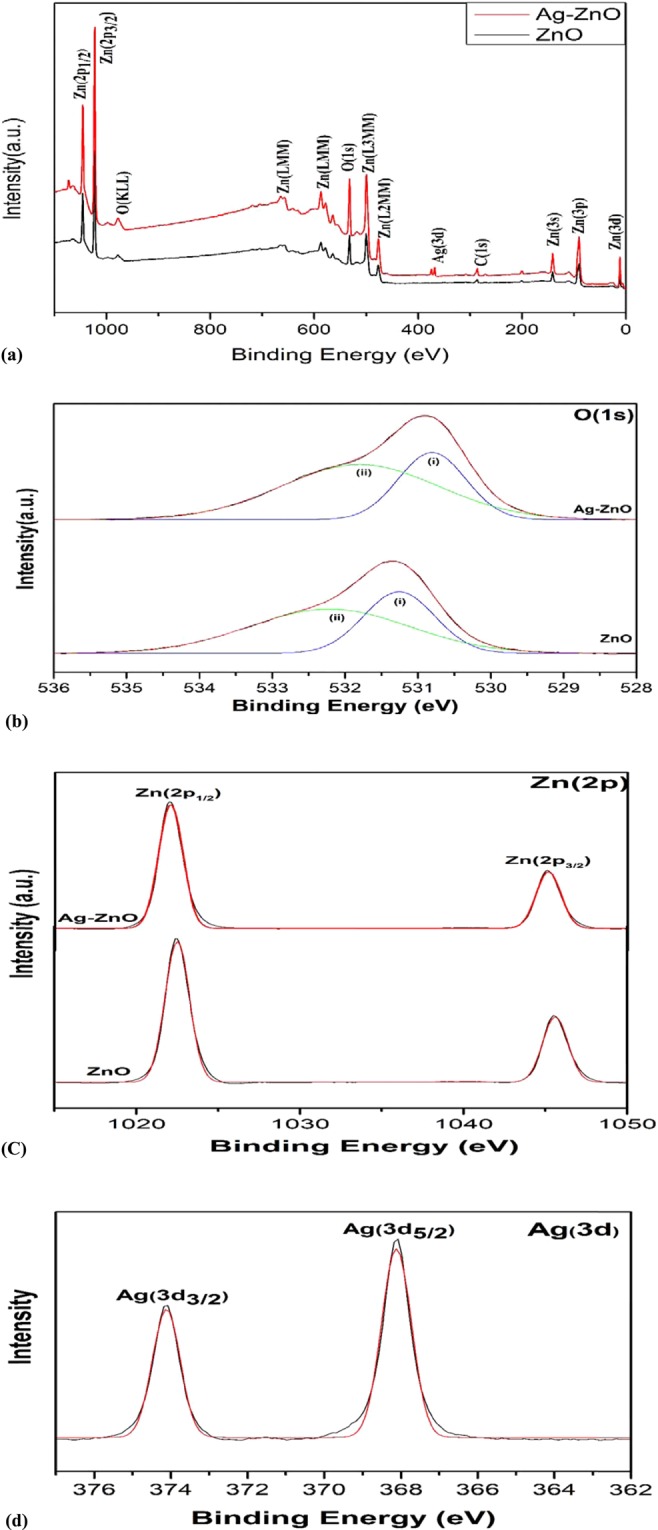
Presents the (**a**) XPS survey spectrum (**b**) O (1 s) spectra (**c**) Zn (2p) spectra (**d**) Ag (3d) spectra.

The determination of elements with chemical bonding states of ZnO and Ag doped ZnO was employed by analyzing XPS spectra shown in Fig. 3.

The full scan spectra shown in Fig. 3(a) identifies the presence of Zn, O, C and Ag in both the samples. The presence of carbon peak C(1 s) at 283.8 eV is presumably due to organic contaminants adsorption on sample surface or acetate vestige^53,54^.

The core-shell level XPS spectra of O (1 s), Zn (2p) and Ag (3d) are shown in Fig. 3(b–d).

In Fig. 3(b) asymmetric O(1 s) curves were fitted with two symmetrical Gaussian curves (i and ii) for both nanoparticles. Peak (i) with lower binding energy in comparison to peak (ii) is attributed to O^2−^ ions of ZnO bonding and adsorption of hydroxyl group which plays a major role in increasing photocatalytic activity by preventing recombination^53^. Silver doping shifted O(1 s) spectrum to lower binding energy which is due to reduced oxygen vacancy^53^.

In Fig. 3(c) two symmetrical peaks at 1022.4 eV,1045.4 eV for pure ZnO and 1022.05 eV, 1045.14 eV for Ag doped ZnO are ascribed to Zn (2p_1/2_) and Zn (2p_3/2_). These peaks are in good agreement with binding energy of stoichiometric ZnO, i.e., 1045.1 eV for Zn (2p_1/2_) and 1022.1 eV for Zn (2p_3/2_) which is attributed to vacancy driven Zn^2+^ to O^2−^ charge transfer

In Fig. 3(d) the Ag (3d) levels are shown for examining the chemical state of Ag element. The Ag(3d_3/2_) peak at 374.08 eV and Ag (3d_5/2_) peak at 368.08 which can be attributed to metallic silver AgO and Ag-Zn-O ternary compound which is consistent with the XRD. Comparatively there is significant shift towards lower binding energy upon doping with binding energy of bulk silver i.e. 368.3 eV for Ag(3d_3/2_) and 374.3 eV for Ag(3d_5/2_) which is because binding energy of unit valency silver (AgO) is much lower than zero valency silver and the formation of oxide layer on surface of silver^53^.

### FESEM

**Figure 4:**
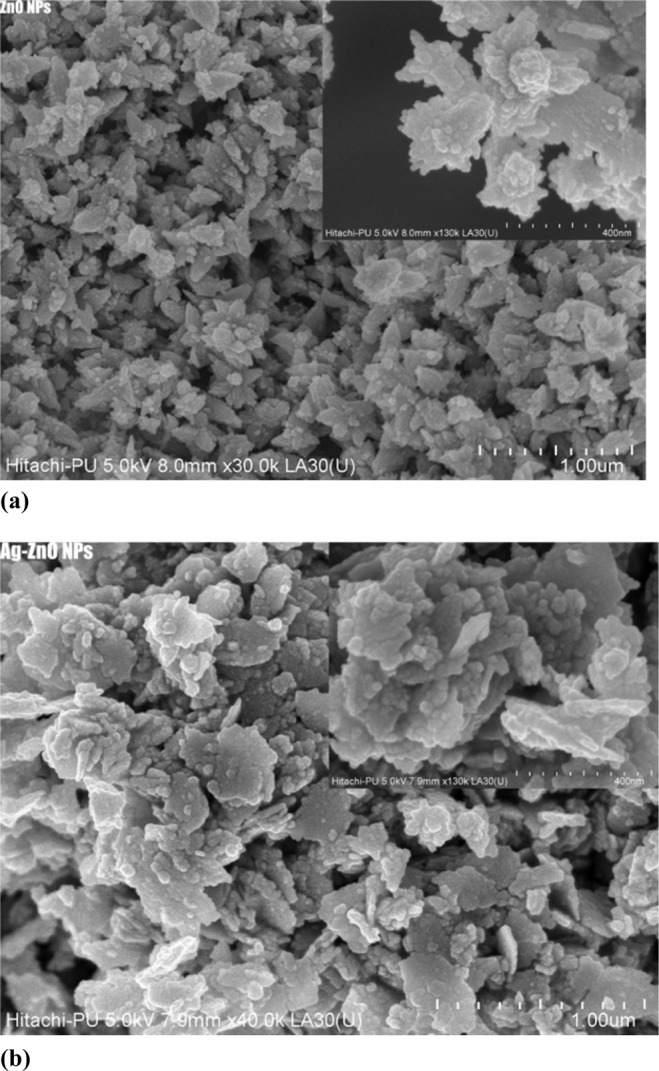
Presents (**a**) FESEM image of ZnO, (**b**) FESEM image of Ag-doped ZnO nanoparticles.

Surface morphology of the samples was investigated through FESEM and are shown in Fig. 4(a,b). Pure ZnO nanoparticles are relatively homogeneous, which can be attributed to the uniform distribution of Zn cations in a three-dimensional structure. The agglomeration is due to densification caused by the narrow space between the particles. For Ag-doped ZnO, the particles are much more agglomerated with the formation of small particles on the larger clusters which may be due to the creation of AgO nanoparticles^55^.

### TEM Analysis

**Figure 5:**
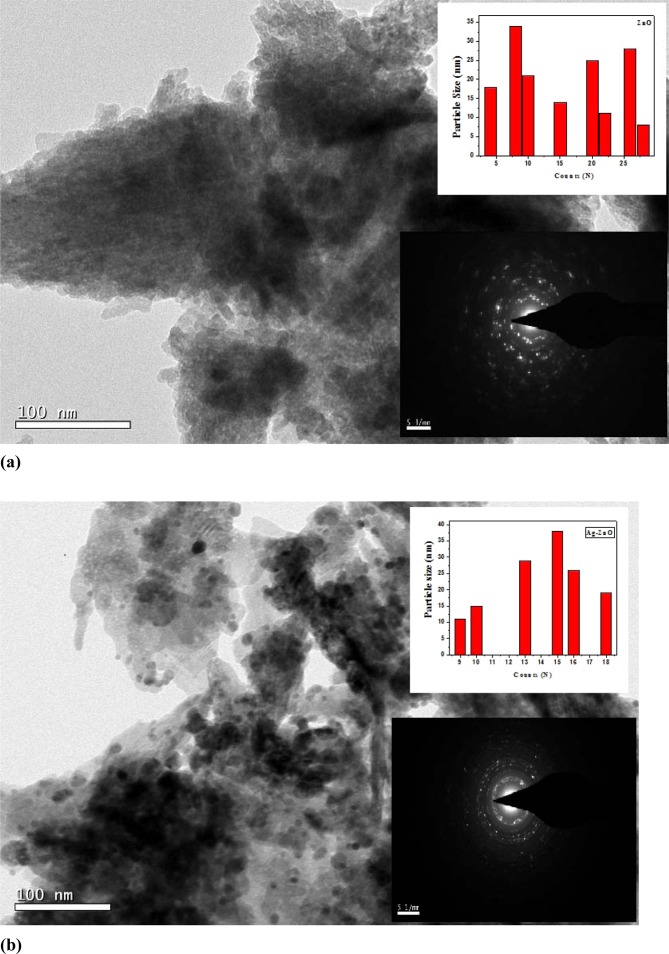
Presents (**a**) TEM micrograph with inset showing particle size distribution and SAED pattern for pure ZnO nanoparticles (**b**) TEM micrograph with inset showing particle size distribution and SAED pattern for Ag doped ZnO nanoparticles.

**Figure 6:**
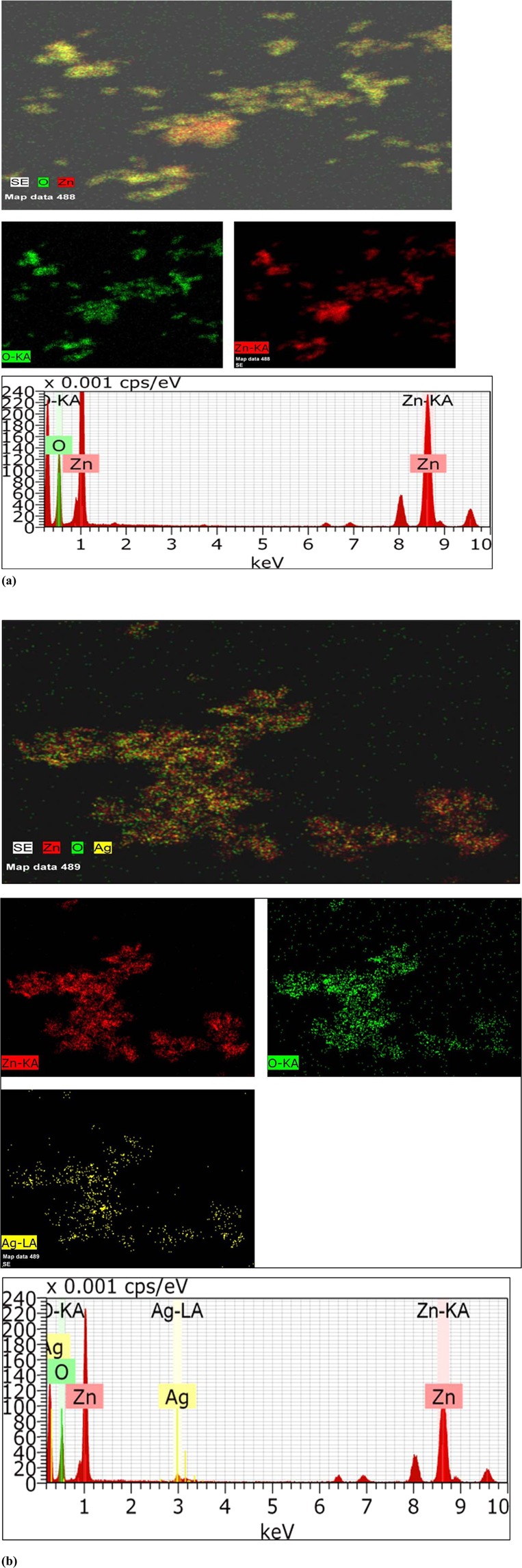
Presents elemental analysis and mapping with EDX (**a**) ZnO (**b**) Ag doped ZnO.

Figure 5(a,b) presents the TEM micrographs of with inset showing particle size distribution as well as the SAED pattern for pure ZnO and Ag doped ZnO nanoparticles, respectively. It is seen that the nanoparticles are highly agglomerated with almost spherical shape. The ImageJ software was used to analyze the micrographs to calculate the particle size as well as to study the shape of the pure and doped ZnO nanoparticles. Particles of 34 nm and 38 nm as maximum has been observed in the TEM of micrographs of pure and Ag doped ZnO. In an SAED, we witness circular ring patterns, which is a signature to confirm the high crystalline order of the samples. In our studied samples, both the samples have shown bright circular patterns, therefore confirming presence of high crystalline order. Figure 6(a,b) presents the elemental mapping of ZnO and Ag doped ZnO nanoparticles using EDS technique. The EDS analysis confirms the presence of all the elements without any additional impurity phase.

### Antibacterial activity

**Figure 7:**
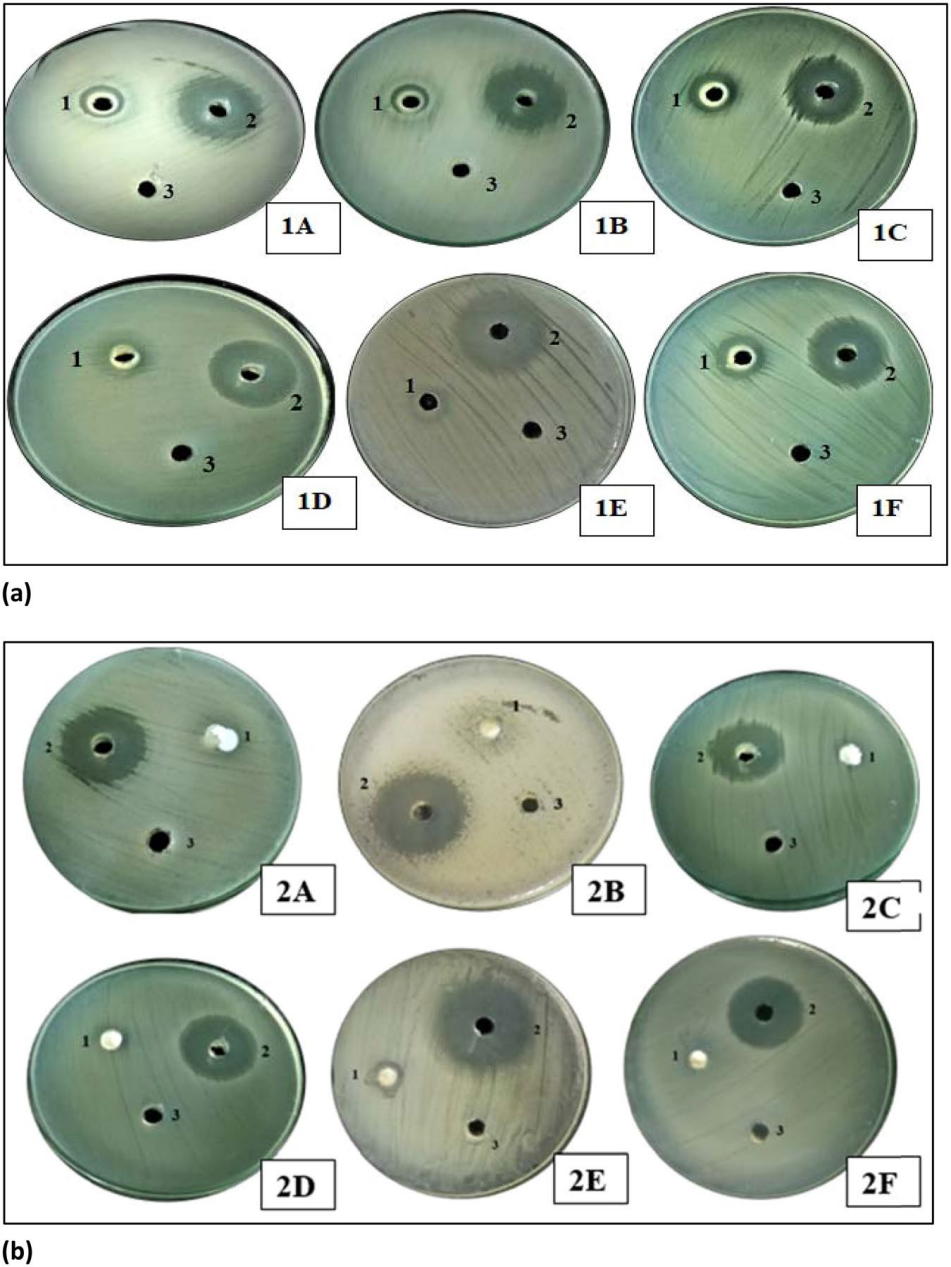
Presents (**a**) Antimicrobial activity of silver doped ZnO(Ag-ZnO) nanoparticles against different pathogenic bacteria; 1A-showed antimicrobial activity against E. coli, 1B-showed antimicrobial activity against Pseudomonas aeruginosa, 1C-showed antimicrobial activity against Staphylococcus aureus, 1D-showed antimicrobial activity against Salmonella typhi, 1E-showed antimicrobial activity against Klebsiella pneumonia, 1F-showed antimicrobial activity against MRSA (Methicillin-resistant Staphylococcus aureus), 1-silver doped ZnO nanoparticles, 2- Positive control, 3- DMSO was a negative control (**b**). Antimicrobial activity of ZnO nanoparticles of cannabis leaves against different pathogenic bacteria; 2A-showed antimicrobial activity against *E. coli*, 2B-showed antimicrobial activity against Pseudomonas aeruginosa, 2C-showed antimicrobial activity against Staphylococcus aureus, 2D-showed antimicrobial activity against Salmonella typhi, 2E-showed antimicrobial activity against Klebsiella pneumonia, 2F-showed antimicrobial activity against MRSA (Methicillin-resistant Staphylococcus aureus), 1- ZnO nanoparticles, 2- Positive control, 3- Negative control. 100 µl nanoparticles were used at a final concentration of 100 mg/ml (prepared in 10% DMSO), 10 µl Positive control for bacteria (Ampicillin) was used at a concentration of 100 mg/ml, Negative control (10% DMSO), produced no zone of inhibition.

The present study revealed that ZnO and Ag-doped ZnO nanoparticles prepared using aqueous leaf extract of *Cannabis sativa* showed potent antibacterial activity against different bacterial strains (Gram-positive and Gram-negative) as shown in Fig. 7(a,b). The inhibition zones (in mm) of varying sizes were obtained as mentioned in Table 3(a,b). The inhibition zones were measured by taking the amount of 100 µl of nanoparticle extract, DMSO and ampicillin in different wells.

**Table 3:** (a,b) presents Inhibition zones of (a) ZnO nanoparticles against *Pseudomonas aeruginosa, Staphylococcus aureus, E. coli*, *Klebsiellapneumoniae, MRSA and Salmonella typhi*. (b) silver doped ZnO(Ag-ZnO) nanoparticles against *Pseudomonas aeruginosa, Staphylococcus aureus, E. coli, Klebsiellapneumoniae, MRSA and Salmonella typhi*.

**a**			
Bacteria	ZnO NPs	Positive control	Negative control
*Pseudomonas aeruginosa*	0	29 ± 0.556	—
*Klebsiella pneumoniae*	9 ± 0.529	28 ± 0.529	—
*Staphylococcus aureus*	10 ± 0.624	10 ± 0.624	
*Salmonella typhi*	10 ± 0.624	23 ± 0.556	—
*MRSA*	8 ± 0.458	25 ± 0.53	—
*E. coli*	10 ± 0.493	29 ± 0.781	—
**b**			
Bacteria	ZnO NPs	Positive control	Negative control
*Pseudomonas aeruginosa*	13 ± 0.458	27 ± 0.655	—
*Klebsiellapneumoniae*	14 ± 0.36	28 ± 0.458	—
*Staphylococcus aureus*	16 ± 0.818	25 ± 0.529	—
*Salmonella typhi*	19 ± 0.556	27 ± 0.655	—
*MRSA*	16 ± 0.404	26 ± 0.721	—
*E. coli*	14 ± 0.568	26 ± 0.656	—

From the above table, it is clearly shown that Ag-doped ZnO nanoparticles indicated the maximum inhibition zone against all pathogenic bacteria as compared to ZnO nanoparticles. Ag-doped ZnO nanoparticles showed maximum zone inhibition against *Salmonella typhi* and minimum zone inhibition against *P. aeruginosa* whereas, ZnO nanoparticles showed maximum zone inhibition against *Klebsiella* and no zone of inhibition against *P. aeruginosa* as shown in Figs. 7(a,b) and 12, 13. DMSO (Negative control) showed no zone inhibition against all bacteria. The ethanol and petroleum extracts of Cannabis leaves showed the inhibitory effects on both Gram-positive and Gram-negative bacteria^55^. The effect of *Cannabis sativa* L. seed oil as well as petroleum ether and methanol extracts of the whole plant on two Gram (+) organisms (*Bacillus subtilis*, *Staphylococcus aureus*), and two Gram (−) organisms (*Escherichia coli*, *Pseudomonas aeruginosa*) have been reported previously^56^. In 2008, Borchardt *et al*. found that the stems and leaves extract was only active against *Staphylococcus aureus*^57–59^.

100 µl nanoparticles were used at a final concentration of 100 mg/ml (prepared in 10% DMSO), 10 µl Positive control for bacteria (Ampicillin) was used at a concentration of 100 mg/ml, Negative control (10% DMSO), produced no zone of inhibition.

### Antibacterial mechanism

**Figure 8:**
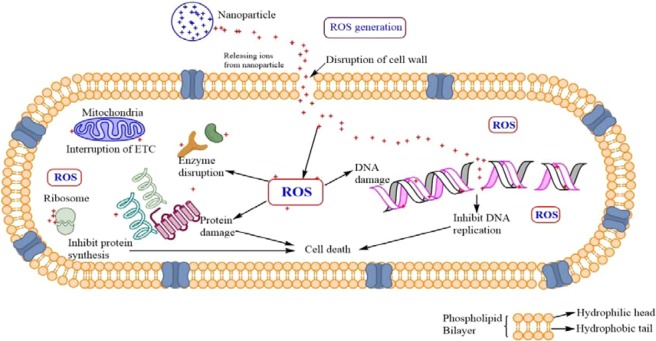
Presents diagrammatic mechanism of antibacterial activity of Ag-ZnO nanoparticles.

Antimicrobial activity against different bacterial strains is represented by ZnO and Ag doped ZnO nanoparticles. Both Gram-positive and Gram-negative bacteria have a negatively charged cell wall, a characteristic that is hypothesized to influence the interactions between the cell walls of the bacteria and NPs or ions released from them. In this study, Zinc oxide releases Zn^2+^ ions whereas, silver release (Ag^+^) ions in aqueous solution contributing to the antimicrobial effectiveness. The released Zn^2+^ and Ag^+^ ions significantly contributed to the overall antibacterial effect of nanoparticles. Nanoparticles have high potential as antibacterial agents attributable to its ability to produce reactive oxygen species (ROS). The generation of reactive oxygen species inhibits the antioxidant defense system, inhibit ATP production and causes mechanical damage to the cell membrane. Recent studies have shown that this ROS generation is profoundly affected by the modification of band structure by introduction of different dopant materials into them. Positively charged NPs, were able to alter the function of the electron transport chain in bacteria. It has been previously proposed that the generation of hydrogen peroxide from the surface of zinc oxide as an effective mean for the inhabitation of the bacterial growth^47,48^. Silver also display antibacterial activity, it can inhibit enzymatic system of the respiratory chain, thus by modifying the DNA synthesis^60^. Different researchers additionally reported that silver nanoparticles enter through the bacterial cell membrane and prompt neutralization of the surface electric charge of the bacterial membrane and change its penetrability, ultimately causing massive cell damage^61,62^. This damage included nuclear fragmentation of the Ag-NPs to the DNA, probably because of the high affinity of Ag^+^ to phosphates highly abundant in the DNA molecule (Fig. 8).

### Anti-fungal activity of nanoparticles

The ZnO and Ag-doped ZnO nanoparticles were also found to be effective against different plant pathogenic fungi. It is essential to mention that the extract of nanoparticles was able to inhibit the growth of fungus (*Fusarium* spp. and *Rosellinia necatrix*) in the present study as well. The inhibition (in mm) of varying sizes were obtained as mentioned in Table 4(a,b) against *Rosellinia necatrix and Fusarium* species. In Ag-doped ZnO nanoparticles, the zone of inhibition of 14.1 mm, 23.25 mm was observed around the nanoparticles and 41.2 mm, 38.3 mm around the negative control against *Rosellinianecatrix* and *Fusarium* spp. respectively. In ZnO nanoparticles, the zone of inhibition of 21.7 mm, 28.2 mm was observed around the nanoparticles and 41.2 mm, 38.3 mm around the negative control against *Rosellinianecatrix* and *Fusarium* spp., respectively.

**Table 4:** Presents Inhibition of pure ZnO and Ag-doped ZnO nanoparticles against (a) *Rosellinianecatrix*. (b) *Fusarium* species.

**a**			
Nanoparticles	*Rosellinia necatrix*	Negative control	% age Inhibition
ZnO nanoparticles	21.7 ± 0.55	41.2 ± 0.6	47.33 ± 0.605
Ag doped ZnO nanoparticles	14.1 ± 0.26	41.2 ± 0.6	65.77 ± 0.230
**b**			
Nanoparticles	*Rosellinia necatrix*	Negative control	% age Inhibition
ZnO NPs	28.2 ± 0.26	38.3 ± 0.44	26.37 ± 0.157
Ag doped ZnO NPs	23.25 ± 0.43	38.3 ± 0.44	39.3 ± 0.626

**Figure 9:**
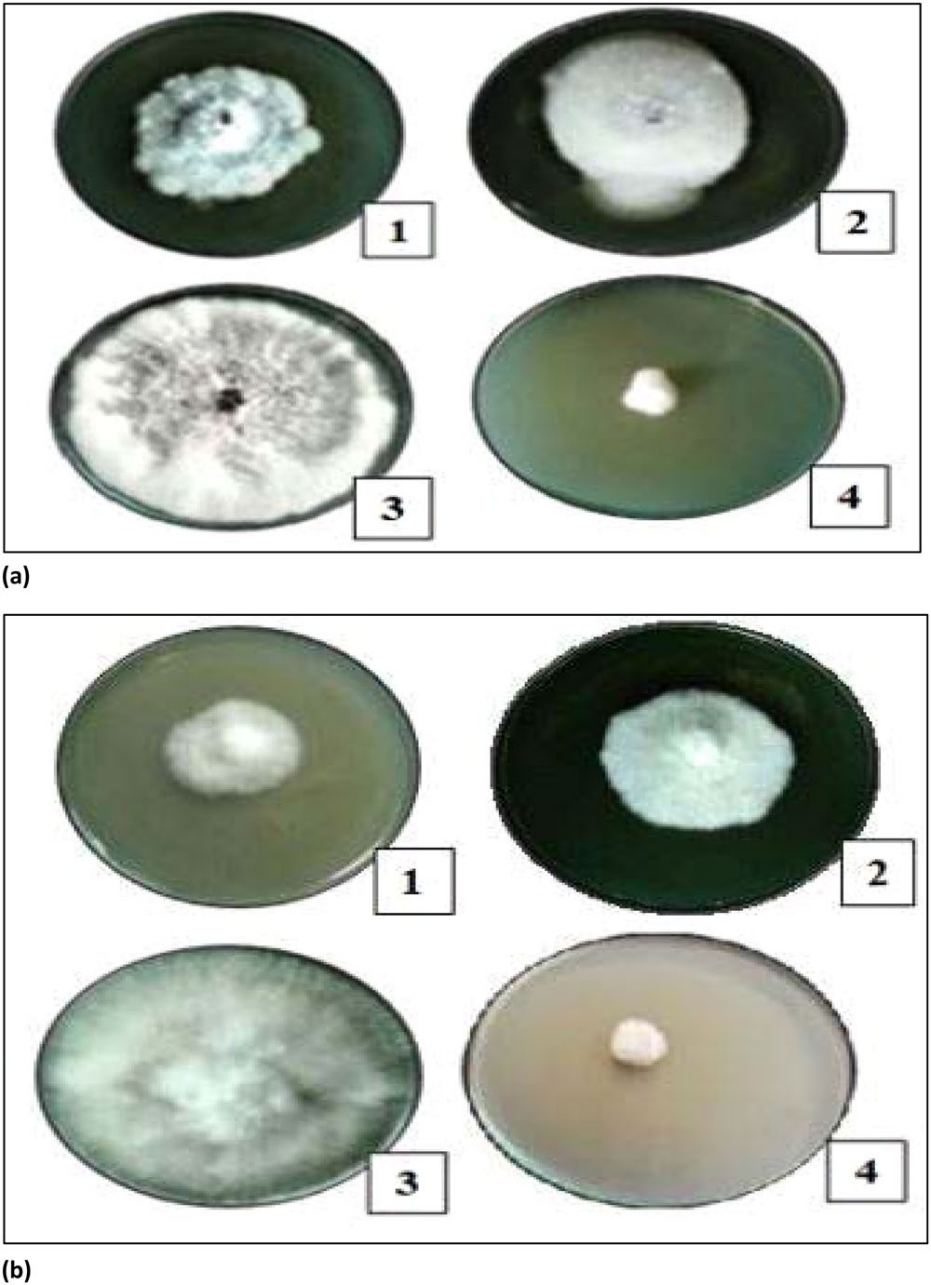
Presents (**a**) Antifungal activity of nanoparticles against Rosellinia necatrix, 1- Silver doped ZnO nanoparticles, 2- ZnO nanoparticles;3-Negative Control; 4-Positive Control, (**b**) Antifungal activity of nanoparticles against Fusarium spp., 1- Silver doped ZnO nanoparticles, 2-ZnO nanoparticles;3-Negative Control; 4-Positive Control.

It is clearly shown that silver doped ZnO nanoparticles indicated the maximum inhibition against *Fusarium* species and *Rosellinia necatrix* as compare to ZnO nanoparticles as shown in Figs. 9(a,b) and 14. The ethanol and petroleum extract of *Cannabis* leaves are effectively inhibiting the growth and development of the collective human pathogenic fungi *Candida albicans* and *Aspergillus niger*, responsible for the black mould in fruits and vegetables^55^. Similarly, the effect of *Cannabis sativa* L. seed oil as well as petroleum ether and methanol extracts of the whole plant on two fungi *Aspergillus niger* and *Candida albicans*^56^ have been reported.

### Photodegradation analysis

**Figure 10:**
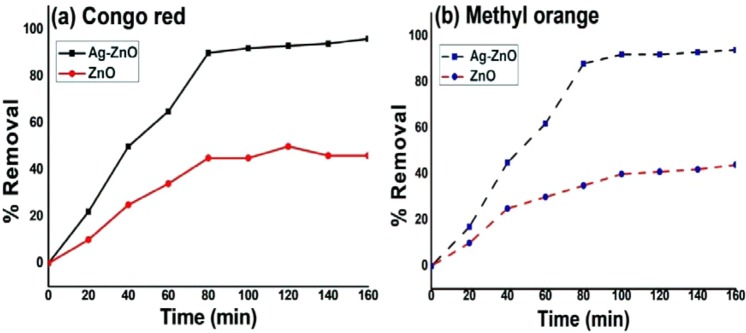
Presents Photocatalytic removal of (**a**) Congo red and (**b**) Methyl orange using Ag-ZnO and ZnO.

Figure 10(a,b) depicts the percentage removal of azo dyes as a function of irradiation time. Nearly, 96% and 38% of Congo red and 94% and 35% of methyl orange were removed using Ag-ZnO and bare ZnO under solar light in 80 min at pH 8. Thus, the removal efficiency of photocatalyst follows the order Ag-ZnO > ZnO. The photocatalytic efficiencies of Ag doped ZnO was remarkably superior as compared to ZnO alone for the removal of azo dye. This was due to considerable adsorption of negatively charged azo dyes at lower pH onto the surface of positively charged Ag doped ZnO.

The phenomena of photodegradation of dyes depends upon the zero point charge of ZnO. The zero point charge (pH_zpc_) of ZnO is 9.0^63^. If the pH_solution_ is higher than pH_zpc_, the surface of ZnO is become negatively charged and at lower pH_solution_ the surface becomes positively charged^64^.

### Mechanism

**Figure 11:**
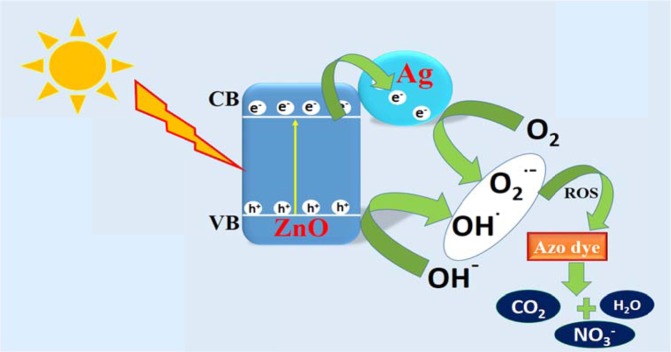
Presents Mechanism of photocatalysis of Ag-ZnO nanoparticles.

ZnO act as photosensitizer and can generate electron-hole pair in the presence of solar radiation. The inefficient harvesting of solar light due to large band gap of ZnO of 3.37 eV and high charge carrier recombination limits practical application of ZnO to be utilized as efficient photocatalyst^65^. Thus, surface modification with a noble metal Ag is done to enhance the photo-efficiency of bare ZnO nanoparticles. As the solar light falls on the surface of Ag-ZnO nanoparticles generation of electron-hole pair take place. These generated charge carriers have very short life span and thus rapidly undergo recombination process which decrease the efficiency of the photo-catalyst. The role of Ag nanoparticle is actually to traps the photo-generated electron by acting as an electron sink and thus averting the charge carrier recombination as shown in Fig. 11^66^. The electrons in conduction band reduces adsorbed oxygen molecule into superoxide radical (˙O_2_¯) and holes oxidizes hydroxide ion into hydroxyl radical (**˙**OH) in the valence band, respectively^67^. The generated reactive oxidation species, oxidizes both congo red and methyl orange azo dye into innocuous molecule.

**Figure 12:**
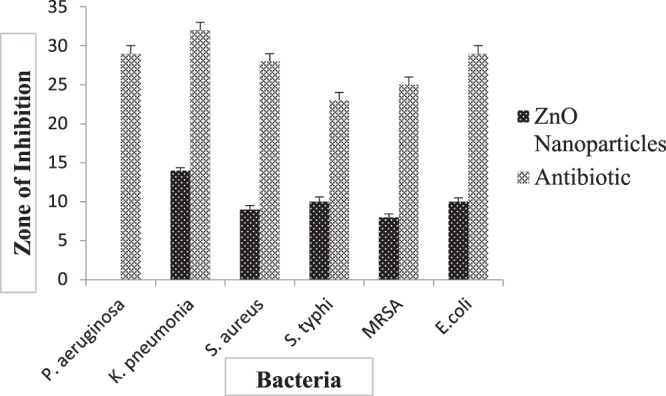
Graphical representation of the antibacterial activity of ZnO nanoparticles against different pathogenic bacteria, *i.e., Pseudomonas, Klebsiella pneumonia, S. aureus, S. Typhi, MRSA (Methicillin-resistant Staphylococcus aureus)* and *E. coli*.

**Figure 13:**
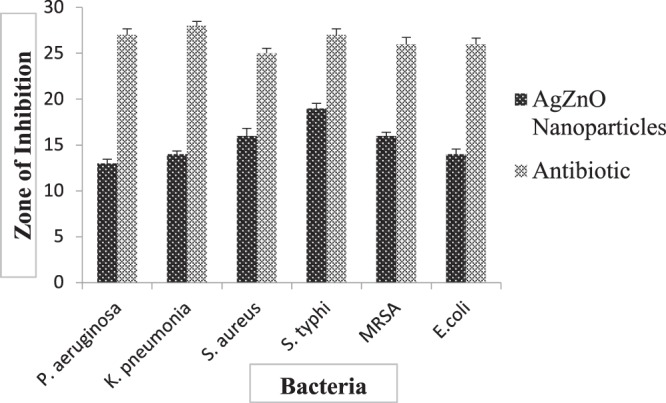
Graphical representation (Mean ± SD) of antibacterial activity of Ag-doped ZnO nanoparticles against different pathogenic bacteria, *i.e., Pseudomonas, Klebsiella pneumonia, S. aureus, S. Typhi, MRSA (Methicillin-resistant Staphylococcus aureus)* and *E. coli*.

**Figure 14:**
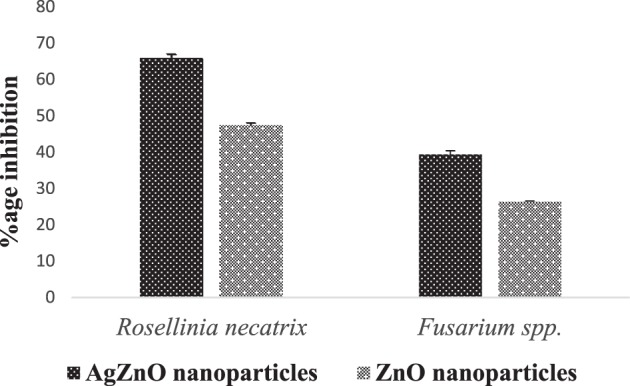
Graphical representation (Mean ± SD) of antifungal activity of ZnO and silver doped ZnO nanoparticles of against *Rosellinia necatrix and Fusarium* species.

The best photocatalytic activity of Ag doped ZnO were found as compared to un-doped ZnO. The deposited metal ions consume the electron and reduces the rate of recombination; thus, majority of electron were utilized to produce superoxide radical in the conduction band rather than recombining with holes.

## Conclusion

Pure ZnO and Ag-doped ZnO were successfully synthesized by an eco-friendly method using *Cannabis sativa* leaves. *Cannabis sativa* used as a reducing and capping agent. The XRD results confirm that silver doping has not altered the structural properties of pure ZnO as have pure hexagonal wurtize structure. The Presence of AgO on the surface of Ag doped ZnO has been confirmed by TEM and XPS. The synthesized Ag-ZnO and ZnO nanoparticles have shown antimicrobial activity against different human pathogenic bacteria (both gram-positive and gram-negative) and different fungal strains. Silver doped ZnO nanoparticles showed the maximum inhibition zone against all bacteria (*Escherichia coli, Klebsiella pneumonia*, MRSA, *Pseudomonas aeruginosa, Salmonella typhi*, *Staphylococcus* aureus) whereas ZnO nanoparticles showed minimum inhibition zone. The ZnO and Ag-doped ZnO nanoparticles were also found effective against two different plant pathogenic fungi (*Fusarium* spp. and *Rosellinia necatrix*) and was able to inhibit the growth of fungi. Ag-ZnO and ZnO nanoparticles removed 96% and 38% of Congo red and94% and 35% of methyl orange under solar light in 80 min. Comparatively, green synthesized Ag-ZnO nanoparticles has shown better results for antimicrobial activity and dye degradation than pure ZnO. Hence green synthesized nanoparticles could have important applications environmental science and biological fields.

## References

[1] Chandran SP, Chaudhary M, Pasricha R, Ahmad A, Sastry M (2006). Synthesis of gold nanotriangles and silver nanoparticles using Aloevera plant extract. Biotechnology progress.

[2] Yadav V (2013). Nanotechnology, big things from a tiny world: a review. AEEE.

[3] Miao L, Shi B, Stanislaw N, Mu C, Qi K (2017). Facile synthesis of hierarchical ZnO microstructures with enhanced photocatalytic activity. Materials Science-Poland.

[4] Qi K (2020). Transition metal doped ZnO nanoparticles with enhanced photocatalytic and antibacterial performances: Experimental and DFT studies. Ceramics International.

[5] Manna J, Begum G, Kumar KP, Misra S, Rana RK (2013). Enabling antibacterial coating via bioinspired mineralization of nanostructured ZnO on fabrics under mild conditions.

[6] Nagajyothi PC (2013). Green route biosynthesis: characterization and catalytic activity of ZnO nanoparticles.

[7] Ozgür, U., Alivov, Y. I., Liu, C., Teke, A. & Reshchikov, M. A comprehensive review of ZnO materials and devices journal of applied physics. (2005).

[8] Ludi B, Niederberger MJDT (2013). Zinc oxide nanoparticles: chemical mechanisms and classical and non-classical crystallization.

[9] Rasmussen JW, Martinez E, Louka P, Wingett DGJE (2010). o. o. d. d. Zinc oxide nanoparticles for selective destruction of tumor cells and potential for drug delivery applications.

[10] Zhang H (2011). A strategy for ZnO nanorod mediated multi-mode cancer treatment.

[11] Seven OZLEM (2004). Solar photocatalytic disinfection of a group of bacteria and fungi aqueous suspensions with TiO2. ZnO and Sahara desert dust.

[12] Tekin D, Tekin T, Kiziltas H (2019). Photocatalytic degradation kinetics of Orange G dye over ZnO and Ag/ZnO thin film catalysts. Scientific reports.

[13] Zare M (2019). Novel green biomimetic approach for synthesis of ZnO-Ag nanocomposite; antimicrobial activity against food-borne pathogen, biocompatibility and solar photocatalysis. Scientific reports.

[14] Qi K, Cheng B, Yu J, Ho W (2017). Review on the improvement of the photocatalytic and antibacterial activities of ZnO. Journal of Alloys and Compounds.

[15] Qi K, Cheng B, Yu J, Ho W (2017). A review on TiO2-based Z-scheme photocatalysts. Chinese Journal of Catalysis.

[16] Liu S, Li C, Yu J, Xiang Q (2011). Improved visible-light photocatalytic activity of porous carbon self-doped ZnO nanosheet-assembled flowers. CrystEngComm.

[17] Giannakopoulou T, Todorova N, Giannouri M, Yu J, Trapalis C (2014). Optical and photocatalytic properties of composite TiO2/ZnO thin films. Catalysis Today.

[18] Yu J, Li C, Liu S (2008). Effect of PSS on morphology and optical properties of ZnO. Journal of colloid and interface science.

[19] Wang S (2019). Direct Z-scheme ZnO/CdS hierarchical photocatalyst for enhanced photocatalytic H2-production activity. Applied Catalysis B: Environmental.

[20] Wu Y (2019). Light-induced ZnO/Ag/rGO bactericidal photocatalyst with synergistic effect of sustained release of silver ions and enhanced reactive oxygen species. Chinese Journal of Catalysis.

[21] Elumalai, K. & Velmurugan, S. J. A. S. S. Green synthesis, characterization and antimicrobial activities of zinc oxide nanoparticles from the leaf extract of Azadirachta indica (L.). **345**, 329–336 (2015).

[22] Harne S (2012). Novel route for rapid biosynthesis of copper nanoparticles using aqueous extract of Calotropis procera L. latex and their cytotoxicity on tumor cells.

[23] Shende, S., Ingle, A. P., Gade, A., Rai, M. J. W. J. o. M. & Biotechnology. Green synthesis of copper nanoparticles by Citrus medica Linn.(Idilimbu) juice and its antimicrobial activity. **31**, 865–873 (2015). 10.1007/s11274-015-1840-3 25761857

[24] Singh A, Singh N, Hussain I, Singh H, Singh SJIJPSI (2015). Plant-nanoparticle interaction: an approach to improve agricultural practices and plant productivity.

[25] Hussain, I., Singh, N., Singh, A., Singh, H. & Singh, S. J. B. l. Green synthesis of nanoparticles and its potential application. **38**, 545–560 (2016). 10.1007/s10529-015-2026-7 26721237

[26] Parveen, K., Banse, V. & Ledwani, L. In *AIP Conference Proceedings*. 020048 (AIP Publishing).

[27] Dhillon, G. S., Brar, S. K., Kaur, S. & Verma, M. J. C. r. i. b. Green approach for nanoparticle biosynthesis by fungi: current trends and applications. **32**, 49–73 (2012). 10.3109/07388551.2010.550568 21696293

[28] Yu J, Yu X (2008). Hydrothermal synthesis and photocatalytic activity of zinc oxide hollow spheres. Environmental science & technology.

[29] Qi K (2014). Geometric Matching Principle for Adsorption Selectivity of Ionic Liquids: A Simple Method into the Fascinating World of Shape‐Controlled Chemistry. Chemistry–A European Journal.

[30] Wang S, Kuang P, Cheng B, Yu J, Jiang C (2018). ZnO hierarchical microsphere for enhanced photocatalytic activity. Journal of Alloys and Compounds.

[31] Lei C, Pi M, Jiang C, Cheng B, Yu J (2017). Synthesis of hierarchical porous zinc oxide (ZnO) microspheres with highly efficient adsorption of Congo red. Journal of colloid and interface science.

[32] Agarwal, H., Kumar, S. V. & Rajeshkumar, S. J. R.-E. T. A review on green synthesis of zinc oxide nanoparticles–An eco-friendly approach. **3**, 406–413 (2017).

[33] Nagarajan, S. & Kuppusamy, K. A. J. J. o. n. Extracellular synthesis of zinc oxide nanoparticle using seaweeds of gulf of Mannar, India. **11**, 39 (2013). 10.1186/1477-3155-11-39 PMC3879036 24298944

[34] Jayaseelan, C., *et al* Novel microbial route to synthesize ZnO nanoparticles using Aeromonas hydrophila and their activity against pathogenic bacteria and fungi. **90**, 78–84 (2012). 10.1016/j.saa.2012.01.006 22321514

[35] Rajiv, P., Rajeshwari, S., Venckatesh, R. J. S. A. P. A. M. & Spectroscopy, B. Bio-Fabrication of zinc oxide nanoparticles using leaf extract of Parthenium hysterophorus L. and its size-dependent antifungal activity against plant fungal pathogens. **112**, 384–387 (2013). 10.1016/j.saa.2013.04.072 23686093

[36] Jacob, S., Bharathkumar, R. & Ashwathram, G. J. W. J. P. R. Aspergillus niger mediated synthesis of ZnO nanoparticles and their antimicrobial and in vitro anticancerous activity. **3**, 3044–3054 (2014).

[37] Samat NA, Nor RMJCI (2013). Sol–gel synthesis of zinc oxide nanoparticles using Citrus aurantifolia extracts.

[38] Gnanasangeetha, D. & Saralathambavani, D. J. A. A. R. J. M. Novel synthesis and characterization of ZnO nanoparticles by Ocimum sanctum. **1**, 164–180 (2013).

[39] Vivekanandhan, S., Schreiber, M., Mason, C., Mohanty, A. K., & Misra, M. Maple leaf (Acer sp.) extract mediated green process for the functionalization of ZnO powders with silver nanoparticles. **113**, 169–175 (2014). 10.1016/j.colsurfb.2013.08.033 24080181

[40] Elumalai, K., Velmurugan, S., Ravi, S., Kathiravan, V. & Ashokkumar, S. (Elsevier, 2015). 10.1016/j.saa.2015.02.011 25725211

[41] Ramesh, M., Anbuvannan, M., Viruthagiri, G. J. S. A. P. A. M. & Spectroscopy, B. Green synthesis of ZnO nanoparticles using Solanum nigrum leaf extract and their antibacterial activity. **136**, 864–870 (2015). 10.1016/j.saa.2014.09.105 25459609

[42] Jiang HE (2006). A new insight into Cannabis sativa (Cannabaceae) utilization from 2500-year-old Yanghai Tombs, Xinjiang. China.

[43] Amar, M. B. J. J. O. E. Cannabinoids in medicine: A review of their therapeutic potential. **105**, 1–25 (2006). 10.1016/j.jep.2006.02.001 16540272

[44] Khan MA, Khan T, Nadhman A (2016). Applications of plant terpenoids in the synthesis of colloidal silver nanoparticles. Advances in colloid and interface science.

[45] Verma, R. *et al* Antimicrobial potential of Ag-doped ZnO nanostructure synthesized by the green method using Moringa oleifera extract. *Journal of Environmental Chemical Engineering*, 103730 (2020).

[46] Murray, P. & Baron, E. (ASM Press, Washington DC, 1995).

[47] Shivapratap H, Philip T, Sharma D (1996). *In vitro* antagonism of Trichoderma species against mulberry leaf spot pathogen, Cercospora moricola. Indian Journal of Sericulture.

[48] Yang H, Ren Y-y, Wang T, Wang C (2016). Preparation and antibacterial activities of Ag/Ag+/Ag3+ nanoparticle composites made by pomegranate (Punica granatum) rind extract. Results in physics.

[49] Mote V, Purushotham Y, Dole B (2012). Williamson-Hall analysis in estimation of lattice strain in nanometer-sized ZnO particles. Journal of Theoretical and Applied Physics.

[50] Wasly H, El-Sadek MA, Henini M (2018). Influence of reaction time and synthesis temperature on the physical properties of ZnO nanoparticles synthesized by the hydrothermal method. Applied Physics A.

[51] Zak AK, Majid WA, Abrishami ME, Yousefi R (2011). X-ray analysis of ZnO nanoparticles by Williamson–Hall and size–strain plot methods. Solid State Sciences.

[52] Karunakaran C, Rajeswari V, Gomathisankar P (2010). Antibacterial and photocatalytic activities of sonochemically prepared ZnO and Ag–ZnO. Journal of Alloys and Compounds.

[53] Hosseini S, Sarsari IA, Kameli P, Salamati H (2015). Effect of Ag doping on structural, optical, and photocatalytic properties of ZnO nanoparticles. Journal of Alloys and Compounds.

[54] Mosquera E (2015). Zinc oxide nanoparticles with incorporated silver: Structural, morphological, optical and vibrational properties. Applied Surface Science.

[55] Oda AM (2015). Study self-cleaning of Congo red from cotton fabric loaded by ZnO-Ag. International Journal of Chemistry.

[56] Wasim K, Haq I, Ashraf M (1995). Antimicrobial studies of the leaf of cannabis sativa L. Pakistan journal of pharmaceutical sciences.

[57] Ali EM, Almagboul AZ, Khogali SM, Gergeir UM (2012). Antimicrobial Activity of Cannabis sativa L. Chinese Medicine.

[58] Sawai J (2003). Quantitative evaluation of antibacterial activities of metallic oxide powders (ZnO, MgO and CaO) by conductimetric assay. Journal of microbiological methods.

[59] Yamamoto O (2001). Influence of particle size on the antibacterial activity of zinc oxide. International Journal of Inorganic Materials.

[60] Brett DW (2006). A discussion of silver as an antimicrobial agent: alleviating the confusion. Ostomy/wound management.

[61] Cho K-H, Park J-E, Osaka T, Park S-G (2005). The study of antimicrobial activity and preservative effects of nanosilver ingredient. Electrochimica Acta.

[62] Jung WK (2008). Antibacterial activity and mechanism of action of the silver ion in Staphylococcus aureus and Escherichia coli. Appl. Environ. Microbiol..

[63] Nadjia L, Abdelkader E, Ahmed B (2011). Photodegradation study of Congo Red in aqueous solution using ZnO/UV-A: Effect of pH and band gap of other semiconductor groups. J. Chem. Eng. Process. Technol.

[64] Bora LV, Mewada RK (2017). Visible/solar light active photocatalysts for organic effluent treatment: Fundamentals, mechanisms and parametric review. Renewable and Sustainable Energy Reviews.

[65] Georgekutty R, Seery MK, Pillai SC (2008). A highly efficient Ag-ZnO photocatalyst: synthesis, properties, and mechanism. The Journal of Physical Chemistry C.

[66] Ren C (2010). Synthesis of Ag/ZnO nanorods array with enhanced photocatalytic performance. Journal of hazardous materials.

[67] Qi K (2013). Morphology-controllable ZnO rings: ionic liquid-assisted hydrothermal synthesis, growth mechanism and photoluminescence properties. CrystEngComm.

